# Genome editing for Duchenne muscular dystrophy: a glimpse of the future?

**DOI:** 10.1038/s41434-021-00222-4

**Published:** 2021-02-02

**Authors:** Christian Kupatt, Alina Windisch, Alessandra Moretti, Eckhard Wolf, Wolfgang Wurst, Maggie C. Walter

**Affiliations:** 1 Klinik und Poliklinik für Innere Medizin I, Klinikum rechts der Isar, Technical University Munich, Munich, Germany; 2 DZHK (German Center for Cardiovascular Research), Munich Heart Alliance, Munich, Germany; 3 Chair for Molecular Animal Breeding and Biotechnology, Gene Center and Department of Veterinary Sciences, and Center for Innovative Medical Models (CiMM), LMU Munich, Munich, Germany; 4 Institute of Development Genetics, Helmholtz-Centre Munich, Munich, Germany; 5 German Center for Neurodegenerative Diseases, Munich, Munich Cluster for Systems Neurology (SyNergy), Munich, Germany; 6 Friedrich Baur Institute, Department of Neurology, LMU Munich, Munich, Germany

**Keywords:** Neurological disorders, Cardiovascular diseases

## Abstract

Mutations in Dystrophin, one of the largest proteins in the mammalian body, are causative for a severe form of muscle disease, Duchenne Muscular Dystrophy (DMD), affecting not only skeletal muscle, but also the heart. In particular, exons 45–52 constitute a hotspot for *DMD* mutations. A variety of molecular therapies have been developed, comprising vectors encoding micro- and minidystrophins as well as utrophin, a protein with partially overlapping functions. With the advent of the CRISPR-Cas9-nuclease, genome editing offers a novel option of correction of the disease-cuasing mutations. Full restoration of the healthy gene by homology directed repair is a rare event. However, non-homologous end-joining (NHEJ) may restore the reading frame by causing exon excision. This approach has first been demonstrated in mice and then translated to large animals (dogs, pigs). This review discusses the potential opportunities and limitations of genome editing in DMD, including the generation of appropriate animal models as well as new developments in genome editing tools.

## The clinical problem of Duchenne muscular dystrophy

New developments of personalized molecular therapy aim at genetically defined disease subgroups in DMD, based on the underlying mutation and the resulting phenotype, and set an example for other hereditary diseases. Although life expectancy of DMD patients has considerably increased within the last decades with the standard use of corticosteroids and non-invasive ventilation, patients become wheelchair-bound in their teens and still die in the course of the disease due to associated cardiac and respiratory complications [1].

Thus, additional strategies are needed to reduce the dystrophic pathology and restore muscle mass and function. These include, but are not limited to, inflammation prevention, muscle growth and regeneration, fibrosis, and improving mitochondrial function. The agents under investigation include a novel steroid [2], NF-κB- [3] and myostatin-inhibitors [4], idebenone [5], an anti-CTGF antibody [6], a histone deacetylase inhibitor [7], and cardiosphere-derived cells [8]. For utrophin modulation, AAV-mediated gene therapy with GALGT2 [9] is currently being investigated to upregulate utrophin expression [10, 11].

Stop-codon read-through is another personalized approach for a DMD subgroup harboring nonsense-stop mutations (~11% of the DMD population), leading to a premature stop of translation, and resulting in a non-functional protein. Aminoglycoside antibiotics, e.g., gentamycin, generate an insertion of alternate amino acids in place of the mutated stop codon, leading to dystrophin production by stop-codon read-through [12]. Since oto- and nephrotoxicity prevent long-term use of gentamycin, Ataluren (Translarna) had been identified as a substance with read-through potential by high-throughput screening [13]. Ataluren is approved in the EU for nmDMD patients aged 2 years and older who are able to walk (https://www.ema.europa.eu/en/medicines/human/EPAR/translarna).

A phase 2a trial showed a mild increase of dystrophin expression [14], a phase 2b trial involving 174 patients aged 5–20 years did not reach significant improvement in the primary endpoint, the 6 min walk test (6MWT). Further analyses indicated that walking ability worsened to a lesser extent with the drug: after 48 weeks of treatment patients could walk on average 32 m more than those given placebo. The effect was more pronounced in a subgroup of patients whose ability to walk was worsening [15]. A phase 3 study in 230 patients aged 7–14 years with worsening walking ability was completed after initial approval, but its results were considered inconclusive. However, data indicated that Ataluren had a positive effect on different measures such as time to run or walk 10 m, time to climb up and down 4 steps and time to loss of walking ability [16]. In both studies, the beneficial effects seemed more evident in patients with moderate decline of their disease. A small study in children aged 2–5 years showed efficacy of Ataluren on an assessment of physical activity in 12 patients when compared with past records of 11 treatment-naïve patients of similar age [17].

Complementing the insight that an internally truncated dystrophin might suffice to exert a therapeutic effect, an exon skipping approach has already been taken to the clinic: Here, the dystrophin RNA was deprived of its mutated exons by interference with the splicing process by antisense oligonucleotide application strategies. These provided a temporary, however detectable expression of a truncated but functional target protein after excision of the mutated exon [18]. The antisense field has recently achieved remarkable progresses with recent accelerated approval of the first antisense oligonucleotide-based therapy for DMD, Exondys 51 [19]. Despite clinical advances, the poor effective delivery to target all muscle remains the main hurdle for antisense drug therapy [20].

Thus, within the last decade, numerous new treatment options made it from bench to bedside; however, there is still a long way to go until these therapeutic strategies will be able to finally cure—and not only modify—pathology and phenotype of DMD patients.

Importantly, a successful therapy would have to start early in development and progression of the disease—between age 2 and 3—to prevent the progressive loss of muscle and motor function. However, in this age group, clinical trials with endpoints such as 6MWT or North Star Ambulatory Assessment are not feasible, resulting in a huge dilemma for the testing of promising substances, and eventually minimizing the effect of the drug due to delayed onset of treatment. In the future, successful treatment of DMD may comprise a cocktail of different drugs and techniques to really make a change toward normal muscle strength and function [10, 11].

## Evolving gene-therapeutic options

Since murine, canine and porcine models of dystrophin mutations are available, novel therapies are intensely studied in the preclinical arena, such as *gene replacemen*t by mini- or micro-dystrophins. The deliberate shortening of dystrophin for therapeutic purposes rests on two elements: first a therapeutic vector with a high degree of myotropism and low toxicity and immunogenicity for systemic application, such as adeno-associated virus (AAV), which has been safely applied in clinical trials. Second, due to the limited packaging capacity of AAV, a shortened form of dystrophin would be required. Here, the original observation that a truncated dystrophin lacking exon 17-48 suffices for normal mobility [21], led to a wide range of mini- and microdystrophin constructs for therapeutic use [22] (cf. the excellent review of D. Duan [23]). For example, minimal dystrophin version lacking spectrin-like repeats (R) 1-24 [24] from the rod domain of dystrophin, but retaining hinges 1 and 4 and the full N- and C-termini, or lacking or R2-15/R18-19/R20-23 and the C-terminus [25], can be packed into an AAV. In the *mdx* mouse model of DMD (a spontaneous mutation introducing a stop codon in exon 23) [26], the former AAV9-based supplementary gene therapy improved cardiac morphology and function, as assessed by histology for fibrosis, by fractional shortening for pump function and by biomarkers indicating heart failure, such as brain natriuretic peptide. In this particular case, a heart-specific promoter was as efficient as an ubiquitously expressing CMV-promoter [24]. In a dog model of DMD (GRMD, point mutation in intron 6 splice acceptor leading to loss of exon 7), the latter approach (ΔR2-15/R18-19/R20-23/C) restored the dystrophin-associated complex, reduced inflammation and fibrosis [25]. More recently, the same GRMD model highlighted the efficacy of an AAV8-microdystrophin (ΔR4– 23/ΔCT) after locoregional (LR) or intravenous (iv) application [27]. The LR application of the therapeutic vector achieved a high rate of transduction (43–59% of the targeted muscle cells), improving function, whereas the iv injection improved the clinical score of GRMD in a dose-dependent manner (with 1 × 10^14^ virus genomes approaching the gait quality of control dogs) [27].

Meanwhile, three companies have developed AAVs encoding microdystrophins: Sarepta uses the ΔR4– 23/ΔCT microdystrophin described above, whereas Solid Biosciences the ΔR2-5/Δ18-2/ΔCT form and Pfizer a ΔR3– 18/ΔCT construct, each under control of a muscle-specific promoter. While clinical trials are ongoing, promising clinical studies aiming at dystrophin expression are progressing, which have the potential to temporarily alter the course of the disease. Their results being eagerly awaited, novel options for gene correction have appeared.

## Animal experiments for *DMD* gene editing therapy: proof of principle and challenges

**Fig. 1:**
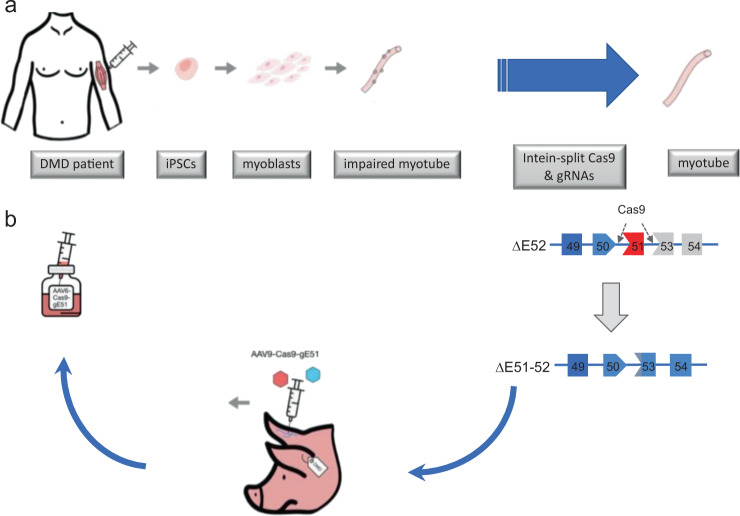
Sketch of the experimental workflow: Patient cells lacking DMD exon 52 were converted to iPS cells and subsequently to muscle cells. An intein-split version of Cas9 was encoded into two AAV vectors, together with two gRNAs excising exon 52. Demonstrating that the AAV-Cas9-gRNAs excising exon 51 (AAV-Cas9-gE51) enabled expression of a shortened but stable dystrophin (51-52, the same approach was successfully used in the porcine model of DMD (lacking exon 52), serving as basis for further development of a therapeutic agent.

As described above, molecular therapeutics enabling expression of a truncated dystrophin have been far developed. However, an unprecedented opportunity to correct the disease-causing mutation has arisen with the advent of Crispr-Cas9 technology (Fig.1).

Since the generation of a Cas9-transgenic mouse [28], which allowed for pinpoint gene alterations specifically in organs targeted by AAVs encoding for the corresponding guide RNAs (gRNAs), it became clear that the inevitable course of inherited diseases might be altered by Cas9-mediated correction. Although certain limitations were unmasked early on, such as the preference of non-homologous end-joining (NHEJ) over homology-directed repair (HDR) upon enzymatic cleavage of the double stranded DNA by Cas9, or the packaging capacity of AAVs, muscular dystrophies seemed an ideal target for genome editing. *DMD* mutations inducing Duchenne muscular dystrophy (DMD) seemed particularly well suited, since internal truncations of the protein may lead to a shortened but stable protein with partial functional restitution and a milder disease progression, as seen in the allelic Becker muscular dystrophy (BMD).

The group of E. Olson was first in showing that correction of the loss-of-function mutation on exon 23 in *mdx* mouse zygotes is possible [29]. Notably, Cas9 combined with a single gRNA was used to inflict a cut in the vicinity of the mutation, accompanied by a single-stranded oligodeoxynucleotide, was efficient in providing HDR in 7 and NHEJ in 4 of the 11 reported corrected *mdx* mice. Whereas HDR correction of 41% of genomes in the mosaic mice sufficed for a full restoration of dystrophin expression in the muscles examined, a 17% HDR correction level yielded a 47–60% of muscle fibers expressing dystrophin, indicating a selection advantage of the corrected muscle and satellite cells. Moving *DMD* correction into the postnatal arena, the same group [30] and others [31–33] demonstrated feasibility of an AAV-based systemic Cas9 treatment, albeit in different flavors. For example, Han and coworkers used a single AAV encoding for *Staphylococcus aureus* (Sa)Cas9 with two guides excising exon 21–23 for systemic delivery of up to 1 × 10^12^ vg in *mdx/Utr*^*+/-*^ mice, showing improvement of muscle force and reduction of fibrosis [33]. In contrast, the other groups used two AAVs containing either Sa- [31, 32] or SpCas9 (*Streptococcus pyogenes*) versions [30] combined with either one [30] or two guides [31, 32] injecting 1.5 × 10^12^ [31], 2 × 10^12^ [32] or 1 × 10^13^ virus genomes [30] systemically. Bengtsson et al. showed efficacy of the AAV6-Cas9-sgRNA transduction improving muscle structure and function in a modified mdx mouse (*mdx* [*4cv*]) harboring a nonsense mutation in exon 53 [34]. In all cases, significant increases of dystrophin expression and muscle function were obtained, indicating that a combination of AAV, Cas9 and appropriate guides may be used to treat DMD by removing the mutated exon and restoring an intact reading frame of the mouse *Dmd* gene.

Large animals, which closer reflect the clinical situation, might pose other hurdles, e.g., with regard to dosage, toxicity and application mode. Of note, the study of Amoasii of the Olson group [35] pioneered a beagle model of DMD. In this model, exon 50 is spontaneously lacking, providing typical features of the human disease such as muscle weakness, atrophy and fibrosis. A combination of 1.2 × 10^13^ vgs of each vector, AAV9-Cas9 and a second AAV9 encoding one gRNA targeting a region adjacent to the exon 51 splice acceptor site (sgRNA-51) [35], sufficed to induce expression of dystrophin in up to 62% after i.m. injection. Systemic application of 1 × 10^13^ and 1 × 10^14^ vgs/kg in one dog each allowed for increasing expression of dystrophin, up to 70% of wild type controls in skeletal muscle and up to 92% in the heart of the higher dosed dog.

Our own group used a porcine model of DMD, inflicted by targeted replacement of *DMD* exon 52 with a neomycin resistance cassette in somatic cells. Nuclei of *DMD*Δ52 pig cells were subsequently used for somatic cell nuclear transfer (SCNT) [36]. Initially the *DMD*Δ52 mutation was introduced into male cells. Thus, all piglets obtained after SCNT were dystrophin deficient and showed clinical, biochemical, and pathological hallmarks of human DMD, but developed them in an accelerated mode [36]. A potential explanation for the highly progressive DMD pathology in pigs, which is associated with characteristic changes of the proteome profiles of skeletal muscle [37] and myocardium [38], is their rapid muscle growth by muscle cell hypertrophy and the associated mechanical strain on the sarcolemma. Since the cloned DMD piglets died before sexual maturity with a maximum life expectancy of 3–4 months, the model could not be propagated by breeding. Chimeric complementation of male *DMD*Δ52 embryos with female wild-type embryos resulted in an adult phenotypic male, which transmitted the *DMD* mutation via its sperm [39]. As an alternative approach, we introduced a heterozygous *DMD*Δ52 mutation in female cells and generated by SCNT a clinically healthy carrier female [36], which was used for breeding with wild-type males to establish a breeding colony. All offspring genotypes, i.e., male DMD pigs, female carriers, as well as male and female wild-type pigs, were obtained at the expected Mendelian ratios of 25% each. Thus, DMD pigs could be routinely provided for gene editing experiments. The model is characterized by a high postnatal mortality (45 of 73 DMD pigs died within the first week). Moreover, none of the animals has survived beyond the 105d timepoint in our study.

### Intein-split Cas9

The out-of-frame mutation inflicted by the absence of exon 52 in our pig model suited well for therapy by an additional Cas9-induced snipping of exon 51, for which two gRNAs were designed and tested in vitro. However, in order to combine it with an AAV vector system, we had to diminish the size of Cas9, similar to the development of microdystrophins. Whereas the packaging capacity of rAAVs allows for a maximum of ~4.7 kbp [40, 41], the human optimized *Streptococcus pyogenes* Cas9 (SpCas9) coding sequence contains already over 4.2 kbp. Combined with promoter and gRNA sequences, the constructs easily surpass 5 kbp, complicating the efficient production of rAAV for carrying the entire CRISPR/Cas9 nuclease system.

Various strategies to reduce the size of SpCas9 were investigated, for example by deleting a 133 amino acids non-essential part of the REC2 lobe. However, this shortened SpCas9 variant retained less than 50% of wild type activity [42]. The application of smaller orthogonal SpCas9 proteins, e.g., like *Staphylococcus aureus (*SaCas9*)* [43], requires more complex PAM sequences which makes it very difficult to identify suitable target sites.

To overcome these limitations and to establish a universal AAV compatible Cas9 expression system, we developed an extein/intein split-version of Cas9 [44]. The general structure of Cas9 in its apo- and RNA/DNA bound holo-form has been characterized in detail. The protein consists of two lobes: a recognition lobe (REC) and a nuclease lobe (NUC). This bi-lobed shaped structure of Cas9 undergoes large conformational re-arrangement upon binding the gRNA/DNA complex [42, 45]. This structural feature renders the rational engineering of Cas9 possible.

Inteins can autocatalytically excise out of a protein and covalently join the remaining flanking regions (exteins) with a peptide bond without leaving a scar [46]. We implemented the extein/intein system of DNA polymerase III DnaE from the cyanobacteria *Nostoc punctiforme* that are located in two genes. N-intein and C-Intein recognize each other, splice themselves out and simultaneously ligate the N- and C-terminal exteins together resulting in a functional full-length DnaE protein [46]. To ensure high splice efficiency, a Cys, Ser or Thr residue is required at the N-terminus of the C-Intein_C-Cas9 fusion [40, 47]. Based on this, for a version (SpCas9 ^[1–573]^ and SpCas9^[574–1368]^) the N- and C-terminal inteins have been introduced between Glu573 and Cys574. In all efficacy-reporting experiments, the split version performed as effective as the wild type Cas9 in respect of genome editing frequency.

### In vitro and in vivo genome editing experiments

Using this AAV9-intein-split Cas9 approach with two gRNAs at 2 × 10^13^ vgs/kg, intramuscular injection revealed a robust, local response with dystrophin protein levels up to 32% of wildtype, which sufficed to improve muscle fiber features such as ferret diameter and proportion of centralized nuclei [48]. A high dose of AAV9-Cas9-gRNA vectors (2 × 10^14^ vgs/kg) broadened the dystrophin expression to the diaphragm and heart, notably significantly shifting the Kaplan–Meier curve of survival rate towards longer survival. Moreover, the mechanism of premature death, which was sudden in nature and without further stigmata of heart failure, appeared to be arrhythmogenic, since the area of low-excitation amplitude was larger in untreated DMD animals and decreased upon high-dose Cas9-gRNA treatment. Moreover, in-depth analysis of calcium signaling in heart slices ex vivo and in AAV9-transduced cardiomyocytes revealed a broadened action potential and an unsynchronized regional excitation of cardiomyocytes cells, respectively.

The porcine experiments were complemented by human induced pluripotent stem cells (iPSCs) from a Duchenne patient carrying a *DMD* exon 52 deficiency analogous to the large animal model. Here, myoblasts derived from untreated iPSCs were unable to further differentiate to myofibers, lacking hallmarks of muscle development such as TTN, MYH1 and CDH15. Upon correction of iPSCs or upon application of AAVs containing Cas9 and human-directed gRNAs, we were able to induce further myocyte differentiation and expression of the sarcomeric proteins named above.

### Economic burden

In the light of emerging new therapies, health economic questions might arise; our group investigated the cost of illness (COI) of DMD and the milder allelic BMD from a socio-economic and clinical perspective in relation to curative or phenotype-modifying therapies [49].

In 363 patients with genetically confirmed DMD or BMD, the consumption of resources of direct medical services, indirect and informal care cost and health-related quality of life (HRQOL) was assessed. Estimated annual disease burden including direct medical/non-medical, indirect and informal care costs of DMD totaled € 65,263, which was nearly twice as high when compared to € 36,651 in BMD. These costs are in agreement with a recent international study comprising patients from Italy, UK and US besides Germany [50]. Here, total annual costs were found in a range of 42,140 $ (Italy), 63,250$ (Germany), 72,870 $ (UK) and 75,820$ (US) in 2012 international dollars. Informal care cost, indirect cost caused by productivity loss and absenteeism of patients and caregivers and non-medical cost were identified as highly important cost drivers. Total cost increased significantly with disease progression and clinical severity of the distinct phenotype regarding all cost items included in the study, whereas health-related quality of life (HRQOL) declined with disease progression.

These health economic assessments are of high importance regarding funding of development programs for rare diseases, since early benefit assessments are required for reimbursement of therapies, and pave the way toward patient access to a new therapy.

### Summary

**Fig. 2:**
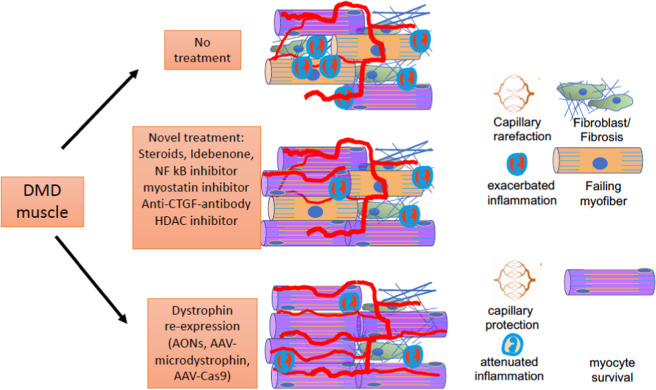
Effects of DMD treatment: DMD muscle fibres without treatment display hallmarks of muscle decay, such as centralized nuclei, fibrosis, capillary rarefaction and continuous inflammation (no treatment). Upon current and novel pharmacologic treatment, inflammation and muscle fibre decay may be decelerated, without alteration of the underlying mechanical strain leading to cell death, e.g., by necroptosis. In contrast, therapeutic strategies aiming at dystrophin re-expression (AONs = antisense olignucleotides targeting exon skipping) correct the disease-causing deficit of dystrophin, either temporally (AONs), or for prolonged intervals (AAV-microdystrophin) or permanently (AAV-Cas9).

Although Duchenne’s muscular dystrophy is a disabling and immobilizing disease with a shortened life span and grave implications with regard to quality of life, the current standard of care has prolonged life expectation by improving care and prophylaxis. A variety of anti-inflammatory and novel pharmacological drugs are studied in clinical trials, such as Vamorolone, NF kB-, myostatin- and histone acetylase inhibitors as well as anti-CTGF antibodies (Fig. 2).

In contrast to these pharmacological approaches, a variety of therapies is aiming at re-expression of dystrophin. As a small molecule, Ataluren is tested in DMD for providing stop-codon readthrough. As antisense oligonucleotide providing exon skipping, eteplirsen gained approval by the FDA. Three different microdystrophins, encoded in AAV vectors, are followed by companies in clinical trials, with results awaited in the coming years.

The latest development comprises the attempt to correct the genetic mutation by AAV-Cas9-gRNA transduction. For that approach, a two-virus system with a suitable Cas9 (being <4.7 kB in size) may be utilized, or a split-intein Cas9, which is distributed evenly with gRNA on 2 AAVs. Both approaches have proven efficacious in large animal models [35, 48]. Off target effects, which were analyzed by gene enrichment and NGS techniques, were not detected. Thus, gene editing by Cas9 and gRNAs for exon snipping may offer a path to future development of a clinically applicable therapeutic option. The development of non-invasive imaging biomarkers, such as multispectral optoacoustic imaging which has been validated in the DMD pig model [51], will support these efforts by allowing longitudinal efficacy monitoring.
